# Assessing Candidacy for Primary Preventative Implantable Cardioverter-defibrillators in Pediatric Patients with Ion Channelopathies: Weighing the Risks and Benefits

**DOI:** 10.19102/icrm.2018.090901

**Published:** 2018-09-15

**Authors:** Madeline L. Townsend, Peter F. Aziz

**Affiliations:** ^1^Department of Pediatric Cardiology, Cleveland Clinic Children’s Hospital, Cleveland, OH, USA

**Keywords:** ICD, implantable cardioverter-defibrillator, ion channelopathy, pediatric

## Abstract

Inherited ion channelopathies have come to the forefront as a significant cause of sudden cardiac death (SCD) in pediatric patients with structurally normal hearts. Implantable cardioverter-defibrillator (ICD) placement can be a life-saving primary preventative therapy, but because of actors inherent in the pediatric population, careful thought must be given to the specific indications for placement in each patient. The most common inherited ion channelopathies are long QT syndrome, Brugada syndrome, and catecholaminergic polymorphic ventricular tachycardia. All have the potential to cause SCD. However, thanks to current research, more is now known about the range of phenotypes present within each disorder and also the benefits that medical therapy can provide. Risk stratification can allow clinicians to best predict which patients may most benefit from a primary preventative ICD while at the same time avoid placement in the larger group who may remain asymptomatic with the aid of medical therapy or even simply observation.

## Introduction

Inherited ion channelopathies have come to the forefront as a significant cause of pediatric sudden cardiac death (SCD) in a structurally normal heart. While the use of an implantable cardioverter-defibrillator (ICD) can be life-saving, it also has the potential to cause significant morbidity, both medically and psychosocially.^1–3^ As such, current research is examining predictors of SCD to help clinicians select those patients most likely to benefit from primary preventive ICD placement.

Furthermore, though ICD placement can be a life-saving primary prevention option, because of factors inherent to the pediatric population, careful thought must be given to the specific indications for device placement in each child. Implanting an ICD in a very young pediatric patient can be technically difficult, and patient growth can precipitate ICD lead compromise.^3,4^ Challenges with programming may lead to inappropriate shocks, either from oversensing, the inappropriate detection of arrhythmias, or ICD “electrical storm.”^5,6^ As with any implanted foreign object, infection is always a risk.^3,6^ Moreover, each specific ion channelopathy carries its own risk profile, with certain complications being more commonly associated with one versus another.

The most common inherited ion channelopathies are long QT syndrome (LQTS), Brugada syndrome (BrS), and catecholaminergic polymorphic ventricular tachycardia (CPVT). All have the potential to cause SCD, and using an ICD as secondary prevention following an aborted cardiac arrest is more often than not standard of care. However, as research advances, the knowledge of how genotypic and phenotypic variation within each disorder affects response to medical therapy has continued to evolve. These data are aiding clinicians to best predict which patients are at the highest risk of having lethal consequences secondary to their arrhythmia disorder. Risk stratification in the pediatric patient with a primary ion channelopathy who is, in some cases, too young to be symptomatic can help to guide treatment options, particularly when it comes to primary preventative ICD implantation **(Table 1)**.^7^

## Common ion channelopathies

### Long QT syndrome

LQTS is a heterogeneous group of ion channelopathies characterized by prolonged ventricular repolarization that may predispose affected patients to life-threatening *torsades de pointes*. The hallmark feature is a prolonged QT interval corrected for heart rate on a 12-lead surface electrocardiogram (ECG). Strong genotype–phenotype relationships have been found for the three main types of LQTS (ie, LQT1, LQT2, and LQT3), with more benign and malignant variants identified.^8–10^ The most common mutations associated with each genotype include LQT1 on *KvLQT1* (alpha subunit on I_Ks_ potassium channel protein), LQT2 on *HERG* (I_Kr_ potassium channel protein), and LQT3 on *SCN5A* (cardiac sodium channel), respectively; all three can cause abnormally prolonged ventricular repolarization.^11^

The protection provided by medical therapy has obviated the need for primary preventative ICD placement in many LQTS patients. ß-blocker medications are first-line therapy and act by preventing a sudden increase in sympathetic activity that can lead to fatal arrhythmias.^10^ They have been shown to significantly decrease arrhythmic events in both LQT1 and LQT2 patients compliant with treatment.^12,13^ Their effect in patients with LQT3 is less certain. Mexiletine, a sodium channel blocker, has been used as a gene-specific treatment in LQT3, with one study showing a significant decrease in annual cardiac events while on therapy.^14^ Left cardiac sympathetic denervation (LCSD) has also been found to be effective and has been used in patients refractory to or unable to take ß-blockers.^15^

**Figure 1:**
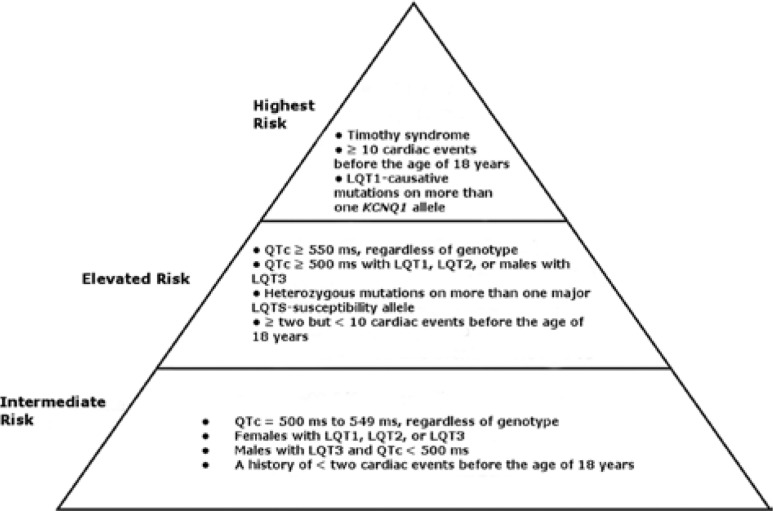
Pyramid of risk stratification for patients at greatest risk of SCD before the age of 40 years without appropriate intervention.^7,8,14,15^ QTc: corrected QT interval.

The “pyramid” of risk stratification for LQTS takes into account the patient’s known genetic mutations, sex, corrected QT interval (QTc), and history of cardiac events **(Figure 1)** and can help to guide management as well as decision-making processes regarding primary ICD placement.^8^ Those who are at the highest risk for SCD before the age of 40 years, without appropriate intervention, include those with LQT1-causative mutations on more than one *KCNQ1* allele; those with ≥ 10 cardiac events before the age of 18 years; and/or those with Timothy syndrome.^16^ Individuals who are at elevated risk include those who have had ≥ two but < 10 cardiac events before the age of 18 years, those with a QTc of ≥ 550 ms regardless of LQTS genotype; those with a QTc of ≥ 500 ms with LQT1, LQT2, or males with LQT3; and/or those with heterozygous mutations on more than one major LQTS-susceptibility allele.^9,17^ Patients at an intermediate risk level include those with a QTc of 500 ms to 549 ms, regardless of genotype; females with LQT1, LQT2, or LQT3; males with LQT3 and a QTc of < 500 ms; and/or those individuals with a history of less than two cardiac events before the age of 18 years.^16,17^

A scorecard (M-FACT criteria^8^) was developed to help determine in which patients ICD implantation might be most appropriate, based on one center’s experience that demonstrated that the majority of patients could be treated effectively without ICD implantation.^18^ Those in whom an ICD should be considered include LQTS patients who have survived cardiac arrest despite adequate ß-blocker therapy of LCSD; those who have survived cardiac arrest off-therapy (except in cases in which preventable cause was identified, including medication-induced or electrolyte imbalance); those who have recurrent LQTS-triggered syncope either despite adequate ß-blockade and cannot have LCSD or with LCSD; and those who are asymptomatic but who have a QTc of ≥ 550 ms with electrical instability and/or a high-risk status despite adequate ß-blockade and LCSD.^19^ This tool in conjunction with patients’ genotype and QT duration can help to risk-stratify patients and prevent the implantation of unnecessary ICDs in those at low risk for SCD.

### Brugada syndrome

BrS is a primary arrhythmia syndrome that is a leading cause of death in Southeast Asian men and is the result of abnormal sodium signaling in cardiac myocytes. Many patients still have undetermined genetic mutations, but, currently, the most commonly found mutation is in the *SCN5A* gene, which encodes the pore-forming subunit of the cardiac sodium channel gene.^20^ Clinical manifestations are relatively rare, with up to 72% of patients being asymptomatic at diagnosis; however, 7% of patients will present with SCD.^21^

**Figure 2:**
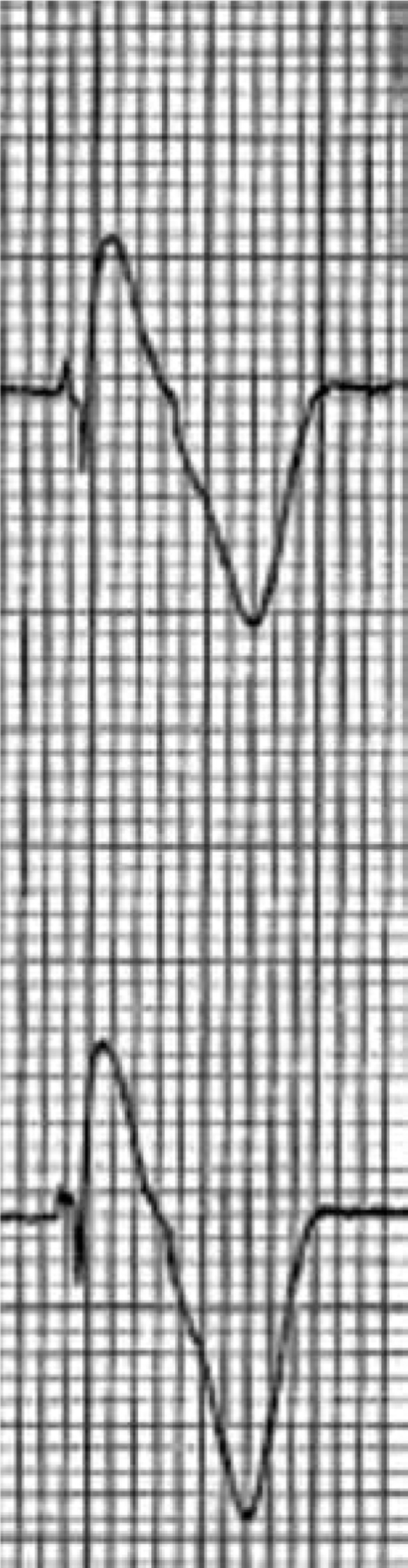
Electrocardiogram (top: V1 and bottom: V2) of a pediatric patient with a type 1 spontaneous Brugada pattern.

BrS has a characteristic ECG pattern of coved-type ST-segment elevation in the right precordial leads positioned in the second to fourth intercostal spaces **(Figure 2)**, unrelated to other factors including electrolyte imbalance, ischemia, or structural cardiac abnormalities. This pattern can be seen spontaneously or following provocative drug testing with class I antiarrhythmic drugs (eg, flecainide, ajmaline) if the baseline ECG was not diagnostic and there is a high degree of clinical suspicion for BrS.

Electrophysiology studies can be performed to try to predict future arrhythmic risk by the inducibility of ventricular arrhythmias in the laboratory, but the prognostic value of these data remain widely debated in the literature^7,22–25^ and there are no known studies specific to the pediatric population. Perhaps the most important value of an electrophysiology study is when it is performed prior to ICD implantation, as evaluating the presence of atrial tachyarrhythmias is important in facilitating ICD therapy programming.^21^

Medications have not been found to be greatly beneficial in BrS, although quinidine may be recommended in patients with a history of arrhythmic storm.^26^ The only proven therapeutic intervention to prevent SCD in BrS is the placement of an ICD; however, every patient with BrS does not necessarily require a primary preventative ICD. Patients at higher risk for SCD include survivors of ventricular fibrillation/SCD; those who have a history of syncopal episodes with spontaneous type I Brugada ECG pattern at baseline; those who are male; and those who have a history of spontaneous atrial fibrillation.^21,27^ Early diagnosis of atrial arrhythmias has also been associated with increased symptoms.^28^ Family history of sudden death has not been shown to translate into increased risk and, so, while the testing of family members is indicated, prophylactic ICD placement in asymptomatic patients is not recommended due to a high risk of complications from the ICD itself.^27^ Recent studies have shown high predictive power for the risk of lethal arrhythmia when the following are present: (1) a history of SCD or syncope; (2) a spontaneous type I Brugada ECG pattern; (3) sinus node dysfunction and/or atrial tachycardia; and (4) conduction abnormality.^21,29^

### Catecholaminergic polymorphic ventricular tachycardia

CPVT is a primary ion channelopathy disease characterized by adrenergic-induced bidirectional and polymorphic ventricular tachycardia (VT). Mutations in genes disrupting intracellular calcium homeostasis in myocytes have been implicated in 60% of cases, and specifically in the genes encoding the cardiac ryanodine receptor 2 calcium release channel and in cardiac calsequestrin.^30,31^ This leads to disrupted sarcoplasmic reticulum calcium release, which can be arrhythmogenic.^32^ Resting ECG is generally normal but, with exercise, ventricular ectopy can develop. Premature ventricular complexes are the initial manifestation, which can then progress into polymorphic or bidirectional VT. Early diagnosis is key, as untreated patients with CPVT have a mortality rate of up to 30% by 40 years of age.^33^ Clinical manifestations are often associated with exercise or emotional stress; one typical presentation might be a 10-year-old with exercise-induced syncope.^34,35^ The occurrence of cardiac arrest prior to diagnosis (but not syncope) and young age at diagnosis are both associated with a higher recurrence risk.^33^

A lack of treatment or compliance with ß-blocker therapy, and specifically nadolol, is an independent predictor for future arrhythmias.^36^ High-dose nadolol is the preferred prophylactic therapy, which requires strict compliance in conjunction with exercise modification. Flecainide was shown in a recent prospective, placebo-controlled crossover clinical trial to reduce the ventricular arrhythmia burden and is now the additive medication of choice when maximal ß-blocker therapy is not sufficient.^37,38^ LCSD has also been used successfully as an antifibrillatory intervention in a few studies.^39,40^

ICD placement should be considered in patients with a history of aborted cardiac arrest but with close care also taken to simultaneously optimize medical treatment. Patients with CPVT experience high rates of inappropriate ICD shocks because of both unwarranted discharges for supraventricular arrhythmias and also spontaneous termination of ventricular arrhythmias.^41^ Research has shown that while ventricular fibrillation is likely to be terminated by appropriate ICD shock, polymorphic VT and bidirectional VT often are not.^41^ Inappropriate discharges have the potential to cause catecholamine-induced surges secondary to pain or fear, which can degenerate into electrical storm or more malignant arrhythmias and have lethal consequences.^41^ Because the pathophysiology of CPVT and the absence of monomorphic reentrant VT obviates the need for antitachycardia pacing, ICD therapy programming in this patient population should include high-rate, single-zone detection for ventricular fibrillation.

## Unique considerations for implantable cardioverter-defibrillators in pediatric patients

One of the major challenges inherent to pediatric patients with ion channelopathies who are being considered for ICD implantation is the determination of programming detection parameters that will correctly identify the malignant arrhythmia and successfully terminate it while also avoiding inappropriate shocks. Specific channelopathies may also have unique goals in programming: for example, permanent pacing can be considered in LQTS to decrease bradycardia-dependent QT prolongation.^26^

Inappropriate shocks are unfortunately common in pediatric patients with primary ion channelopathies. One study found that, in a group of 76 patients aged younger than 30 years of whom 33% had primary electrical disease, 19 patients (25%) received inappropriate therapy. The unwarranted shocks resulted from multiple causes, including lead failure (seven patients), sinus tachycardia (eight patients), supraventricular tachycardia (four patients), and T-wave oversensing (two patients).^6^ Lead failure was the most common chronic complication, occurring in 21% of patients, with those patients who were smaller and/or younger at greater risk for this complication.^6^

Another study that evaluated the rate of inappropriate shocks in 144 pediatric and congenital heart disease patients found that 9.7% (14 patients) received an inappropriate shock and that the occurrence of such was related to a lower detection rate programmed in the VT zone.^5^ This study concluded that programming in a higher detection rate and a longer detection duration could result in a lower rate of inappropriate shocks, without associated adverse effects, in a pediatric population.

In addition to challenges in ICD programming, ICD placement and maintenance can be problematic in pediatric patients. Because individuals with primary ion channelopathies do not typically require pacing, one potential solution to the lead complications caused by physical growth could be found with subcutaneous ICD (S-ICD) placement.^42^ In one small study, pediatric patients were case-matched with S-ICDs or transvenous ICDs: the results indicated there was a similar survival benefit between the two groups but a lower rate of complications requiring reoperation in the S-ICD group.^43^ However, an S-ICD does have the potential to oversense high T-wave voltages as another study has found, with up to 13% screening failure occurring in patients with S-ICDs and an inherited primary arrhythmia syndrome.^44^

## Conclusion

The selection of appropriate pediatric patients affected by an ion channelopathy as candidates for primary preventative ICD implantation remains a challenge that requires risk stratification based on symptoms, past medical history, known genetic defects, and compliance with medical treatment. An ICD has the potential to abort SCD, but care must also be taken to provide maximal medical therapy as well as counseling on lifestyle changes and at-risk situations for these patients. Morbidity from ICD placement can include ICD lead fracture, infection, and inappropriate shocks, which can in turn increase psychological stress, medical illness, and health care costs. With continued improvements in ICD manufacturing and programming, one can hope that morbidity secondary to an ICD will continue to decrease. Moreover, medical therapy can have a significant role in treatment and preclude the need to place a primary preventative ICD in a select group of patients. More pediatric-specific data are needed to allow clinicians to accurately risk-stratify young patients with ion channelopathies and to facilitate the selection of those individuals who will most benefit from primary preventative ICD placement, while at the same time avoiding placement in the larger group who may remain at low risk for SCD with the aid of medical therapy or even simply observation.

## Figures and Tables

**Table 1:** ICD Placement Recommendations in Patients with Ion Channelopathies per the 2013 Heart Rhythm Society/European Heart Rhythm Association/Asia Pacific Heart Rhythm Society Expert Consensus Statement^19^

Disease	ICD Placement Recommendations	Class of Evidence
Long QT syndrome	Recommended for survivors of a cardiac arrest	Class I
	Consider for patients who have recurrent syncopal events while on ß-blocker therapy	Class IIa
	Not indicated in asymptomatic patients not tried on ß-blocker therapy	Class III
Brugada syndrome	Recommended for survivors of a cardiac arrest and/or those who have documented spontaneous sustained VT with or without syncope	Class I
	Consider in patients with spontaneous diagnostic Brugada type I ECG pattern who have a history of syncope judged to be likely caused by a ventricular arrhythmia	Class IIa
	Consider in patients who develop VF during programmed electrical stimulation (inducible)	Class IIb
	Not indicated in asymptomatic patients with drug-induced Brugada type I ECG pattern and on the basis of family history of SCD alone	Class III
Catecholaminergic polymorphic ventricular tachycardia	Recommended in patients who experience cardiac arrest, recurrent syncope, or polymorphic/bidirectional VT despite optimal medical management and/or LCSD	Class I
	Not indicated as the sole therapy in an asymptomatic patient	Class III

ICD: implantable cardioverter-defibrillator; VT: ventricular tachycardia; ECG: electrocardiogram; VF: ventricular fibrillation; SCD: sudden cardiac death; LCSD: left cardiac sympathetic denervation.
